# Teaching the Concept of Computational Thinking: A STEM-Based Program With Tangible Robots on Project-Based Learning Courses

**DOI:** 10.3389/fpsyg.2021.828568

**Published:** 2022-01-27

**Authors:** Ming-Chia Hsieh, Hui-Chun Pan, Sheng-Wen Hsieh, Meng-Jun Hsu, Shih-Wei Chou

**Affiliations:** ^1^Department of Marketing and Logistics Management, Far East University, Tainan City, Taiwan; ^2^College of Management, National Kaohsiung University of Science and Technology, Kaohsiung City, Taiwan; ^3^General Education Center, National Tainan Junior College of Nursing, Tainan City, Taiwan; ^4^Department of Hotel Management, National Kaohsiung University of Hospitality and Tourism, Kaohsiung City, Taiwan; ^5^Department of Information Management, National Kaohsiung University of Science and Technology, Kaohsiung City, Taiwan

**Keywords:** computational thinking (CT), tangible robots, project based learning (PBL), cognitive load, learning performance (LP)

## Abstract

The twenty-first century is arguably the century of computing. In such a world saturated by computing, Computational Thinking is now recognized as a foundational competency for being an informed citizen and being successful in STEM work. Nevertheless, how to effectively import different types of teaching methods in university courses (lecture based learning, project based learning) is subjected to further evaluation. Currently, the arguments in favor of tangible robots including high interaction, great practicality, and specific operation results make themselves to be often used as a teaching medium and tool for teaching activities between teachers and students. Hence, in addition to cultivating students with computational thinking ability, this paper discussed how to integrate tangible robots into project-based learning courses of thinking skills training to improve the learning performance of the computational thinking ability. This study conducted in one semester on the 105 students from three classes. Experimental results show that the project-based learning method integrated with the teaching material of robotic visual programs approach had significantly better effectiveness in improving students' learning achievements than the traditional teaching method integrated with paper practice teaching materials approach. Analysis of the questionnaire results showed that the proposed learning approach did not increase the students' cognitive burden. In sum, the proposed approach helps students' learning achievement and cognitive load.

## Introduction

The twenty-first century has been said to be a computing century (Grover and Pea, 2018), with applications such as artificial intelligence, big data, speech and facial recognition, robotics, Internet of Things, cloud computing, autonomous vehicles and other technologies integrated into many aspects of life. This is changing the ways people work, interact, communicate, shop, dine, travel, and get news and entertainment (Grover and Pea, 2018). In a world filled with computing behavior, computational thinking (CT) had been recognized as a basic capability to succeed in STEM-related work (Bertrand and Namukasa, 2020; Bai et al., 2021; Lin et al., 2021). The concept of computational thinking was proposed by Wing (2006) as an analytical approach to thinking based on computational concepts. It is a basic capability to solve problems, design systems, and explore human behavior. Taylor and Baek (2019) contended that computational thinking allowed people to use a thinking mode similar to that used by computer scientists when facing problems. With the rise of automation and artificial intelligence, economists predicted there would be 800 million relevant jobs available by 2030 (McKinsey Global Institute, 2017). Therefore, it is very important to train students with good computational thinking abilities to help them face this technology-driven world (Voogt et al., 2015; Wing and Stanzione, 2016; Tsortanidou et al., 2019; Fidai et al., 2020; Tang et al., 2020; Kaspersen et al., 2021; Lavi et al., 2021; Israel-Fishelson and Hershkovitz, 2022).

As part of this, countries worldwide have made substantial investments in policies developing intelligent robots for industry, and robots for use in education are an area worthy of development. Educational robots act as teachers to transmit educational material or help students to better understand the educational content, and this is referred to robot teaching (Benitti, 2012). The traditional teaching mode focuses on collective teaching and is relatively easy to implement teaching methods. Traditional teaching is based on teacher-based lectures, with less time for students to participate and fewer opportunities for interaction between teachers and students. Educational materials were from teachers or teaching material prepared by them. It also has limited ability to assess and diagnose students' learning, but waiting until testing. In contrast, robots in the classroom teaching can provide high interactivity, practical operation, flexible operations, and evaluation of results. It can both align with students' cognitive ability, and stimulate their learning motivation with novel robotic teaching materials (Yin, 2021).

The process of knowledge development is based on senses, including of vision, hearing, touch and kinesthetics (Zager et al., 2012). Through the accumulation of experience gained from these perceptions, knowledge could be internalized. One of the main differences between instruction provided by a tangible robot and that of traditional teaching is that the senses used in traditional teaching activities are only visual and auditory. In contrast, when using a tangible robot as a teaching medium, its touchability can supplement the visual and auditory senses, enhancing learning effect (Lemay et al., 2021). These multi-sensory teaching methods can enhance learning interest, concentration, memory, and emotional focus to improve learning achievement.

With advances in robotics, robots can improve the learning motivation of students (Hung et al., 2013), though the integration of robots in daily teaching activities is still limited. Therefore, this paper seeks to integrate them into courses of computational thinking. Tangible robots were deployed to provide learners with different sensory learning preferences and thereby enhance their learning. Hence, in addition to cultivating students' computational thinking capabilities, this research also explores how to integrate tangible robots computational thinking courses. An experiment was conducted to demonstrate the effectiveness of the proposed approach.

## Literature Review

### Computational Thinking

As Wing (2006) defined computational thinking, it is a thinking model using basic concepts of computer science for problem solving, system design and understanding human behavior. She contended that computer computing should be a factor added to basic language skills. In addition to reading and writing, computational skills should be added into the concepts of computer science. The thinking skills of computer science were not merely to obtain patents for computer scientists, but also should be part of the capabilities and literacy necessarily for anyone. Wing and Stanzione (2016) further proposed computational thinking was not only about problem solving, but also about a series of abilities in problem formulation. Computational thinking (CT) (Wing, 2006) was proposed as the basic ability to become an informed citizen, making STEM-related work successful (Grover and Pea, 2018). Kao (2011) proposed four aspects of computational thinking based on Wing (2006)'s proposals. According to these four dimensions, BBC Bitesize (2017) revised the pattern generalization to re-propose four aspects of computational thinking for abstraction. These second four aspects were also adopted by Google as the content of computational thinking.

### Project-Based Learning

Project-based learning (PBL) originated from medical education in the 1960s, based on Dewey's progressive educational theories (Marx et al., 1997). PBL courses increase the interest of students by providing them with opportunities to deal with contextualized problems, connecting what they learn in schools with their experiences outside school (Brown et al., 1989; Jurow, 2005). Bell (2010) contended that PBL created key strategies for independent thinking and learning. PBL could not only teach what students should learn, but also how students should learn (Wilhelm et al., 2008). Learners to submitted results that allowed them to find problems in topics using problem-solving methods and active learning. From learning tasks, learners could construct a teaching method based on their own knowledge (Thomas, 2000), thereby enhancing students' involvement in learning (Guo et al., 2020). As project-oriented learning was verified, it gave students the abilities of active learning, pursuing their interests and solving problems (Duke et al., 2021).

In addition to medical education, PBL pedagogy was also applied in computer science. The University of Sydney in Australia found that students' initial skills and attitudes toward information science would affect their future learning performance (Bell, 2005), so PBL pedagogy was used in courses on programming languages in 1996. The University of Delaware began to adopt the PBL pedagogy model in physics, chemistry and biology courses two decades ago (Herreid, 2003).

Preparing PBL teacher's guides for project-based learning is a complex task (Fleming, 2000). Based on difficult and challenging problems, learners work on designing processes, solving problems, making decisions, investigation and research. These activities cultivate independent thinking and show how to make things work and complete tasks. The current study applies PBL in an initial stage, a development stage and an evaluation stage. The initial stage includes driving problems, basic teaching and guiding students to find resources. The development stage includes project planning and production. The evaluation stage includes self-evaluation, self-correction, achievement sharing and evaluation.

### Tangible Robot

Robots can do highly repetitive or dangerous tasks that humans are unwilling to do, or work under conditions not suitable for humans, like outer space or the deep sea. With the development and integration of technologies such as mechanics, electronics, information and big data, the functions and technical levels of robots have been greatly improved, with their applications in various industries becoming increasingly mature. In particular, the production of intelligent robots has extended from automatic equipment such as mechanical arms to humanoid or doll-like robots. Robots have also been integrated into teaching applications and personalized services, and applications combining robots with artificial intelligence technologies could greatly extend their use.

The continuous innovation of robot researches and technological development could also have considerable impact on the educational environment, especially in the current K-12 learning environment. Educational robots have already been used to assist special-education learning activities (Benitti, 2012; Hung et al., 2013; Taylor and Baek, 2019; Witherspoon and Schunn, 2019; Kucuk and Sisman, 2020; Sen et al., 2021; Yin, 2021). From a market perspective, the definition of educational robots could be divided into two types of products. First, educational robotics provide a learning environment motivating participants to design and build robots, basically a teaching aid used for robot education. For example, Lego and Mbot could stimulate the creation of robot products. In the second category, educational robots indicate a complete robot product functioned with teaching intelligence educational activities. For example, NAO robots could be used to accompany with children for learning. The educational robots indicated in this research belong to the second category, functioning with teaching and dialogue skills.

### Cognitive Load

The cognitive load theory proposed by Sweller (1988) has been used to construct human cognitive structure models and to develop teaching design principles and strategies (Van Merriënboer and Sweller, 2010). Sweller et al. (2007) defined cognitive load as a kind of “cognitive energy” meaning the interaction between mental load and effort when people face the load generated by messages (Sweller, 1988). Cognitive load theory can also provide the basis for developing instructional design (Kalyuga and Sweller, 2004; Sweller et al., 2007; De Jong, 2010). For the design of instructional content, Sweller (2021) contended that instructional design was required for the processing of working memory. The sources of learners' cognitive load are divided into three types: intrinsic cognitive load, extraneous cognitive load and germane cognitive load (Van Merrienboer and Sweller, 2005).

Subjective rating scales (Leppink et al., 2013) are the most commonly used to measure cognitive load in the past. For example, the 7-point mental effort scales proposed by Sweller et al. (1998) are recognized as a credible cognitive load measurement method.

## Research Design

This study uses quasi-experimental design methods to explore how different teaching methods for computational thinking affect the computational thinking and utilization abilities of university students. It considers how different teaching methods influence computational thinking abilities of university students.

### Participants

Freshman students from a private university of science and technology in Tainan City were recruited as experimental subjects. There were 105 students recruited and randomly assigned into three groups (control group, experimental group A and experimental group B). The 35 students in the control group received traditional teaching methods. The 35 students in experimental group A received project-based learning methods integrated with a computer-based visual program. The 35 students in experimental group B receiving project-based learning methods integrated with a robot-based visual program. To avoid randomly assigned students having significant background differences, the random assignment process used sampling so that the groups were balanced for IT-related majors and for gender.

### Measuring Tools

To understand how different teaching methods influence the learning effectiveness of students' computational thinking, Bebras (International Challenge on Informatics and Computational Thinking, http://www.bebras.org) was used as a test tool to analyze the improvements in computational thinking capabilities of the students from different experimental groups.

Scoring of the Bebras questions for international computing thinking capabilities was based on the difficulty of individual questions. Points were added for correct answers and deducted for wrong answers. No points were added or deducted for questions not answered. There were 15 questions for every test and different difficulty levels were equally assigned into 5 questions on average. Easier questions would add 12 points for correct answers, but deduct 3 points for wrong answers. Moderately difficult questions would add 16 points for correct answers, but reduce 4 points for wrong answers. Questions with difficult levels would add 20 points for correct answers, but deduct 5 points for wrong answers. The overall initial score was 60 points. The lowest score was 0 points and the highest score was 300 points.

The cognitive load questionnaire was developed based on the cognitive load measure proposed by Sweller et al. (1998). It consisted of 4 questionnaire items, each with a 7-point Likert scale. The Cronbach's alpha of the questionnaire was 0.88, showing adequate internal consistency in evaluating the cognitive load of the students.

### Experimental Procedure

The experiment was meant to understand the effectiveness to enhance computational thinking capabilities among university students derived by different teaching methods of computational thinking. Table 1 shows the research design. In line with experimental work, we regarded the teacher, classroom environment, and teaching materials as control variables. We invited freshman in a general education course of computer concepts to participate in the experimental study. Then, the students were randomly divided into three groups. We tried to understand the student responses' characteristics *via* the simple oral report, showing their academic background non-related to IT or engineering field, and the less experience of using robotics.

**Table 1:** Experimental procedure of three groups.

**Group**	**Pre-test**	**8 weeks of teaching**	**Post-test**	**Followup-test**
	**(week 1)**	**(week 2**~**9)**	**(week 10)**	**(week18)**
Experimental group A	O_1_	T_P+S_	O_2_	O_3_
Experimental group B	O_1_	T_P+R_	O_2_	O_3_
Control group	O_1_	T_L_	O_2_	O_3_

*O_1_: The pre-test adopted the Bebras questions (an official version); O_2_: the post-test adopted the Bebras questions (a modified version A); O_3_: the followup-test adopted the Bebras questions (a modified version B); L: the traditional teaching method; P: project-based learning method; S: the computerized graphical program “Scratch”; R: the graphical program module entity was combined with tactile NUWA Robotics*.

Before experiments, during the first week, the Bebras questions (an official version) was used to conduct a pre-test (O_1_) on three groups of students to explore the basic degree of students' computational thinking capabilities and understand the starting behavior of students' various computational thinking elements. In the teaching experiment processes, three classes taking the required general thinking and training courses were integrated into 8-week teaching courses and 2-week 120-min teaching courses of computing thinking capabilities.

The control group used teachers for traditional teaching with slides. It was further integrated with the book (Scratch programmed by Program the World) to conduct an unplugged teaching method (L). Teaching contents and methods were mainly conducted by traditional didactic teaching, aided by student practicing.

Experimental group A adopted a project-based learning method (P). Teachers acted as learning assistants or coaches to provide necessary learning guidelines and resources. Students were requested to research a topic or theme. These research questions originated from daily life. Computer classrooms allowed students to use the Scratch (https://scratch.mit.edu/) for topic development (S).

Experimental group B also adopted a project-based learning method (P), with teachers acting as learning assistants or coaches to provide necessary learning guidelines and resources. Students were requested to research a topic or theme. These questions also originated from daily life. Moreover, the classroom environment allowed students to develop special topics for tangible NUWA (https://www.nuwarobotics.com/en/) Robotics (R) shown in Table 2. NUWA is a STEM-based robot program that gives student real-time support, including a computer monitor screen that displays learning materials, videos and games, bringing interactivity to users. We afforded NUWA for every individual participants in the experimental context. STEM is an interdisciplinary and applied approach (Bertrand and Namukasa, 2020). In this study, our STEM-based robot programs depend on the idea of students in multi-sensory teaching methods, its touchability can support the visual and auditory senses, enhancing learning interest and constantly focus on projects. Eventually, improving the STEM experience for student is aimed to our programs, and help students achieve their potential of computational thinking ability as learners by the robot's assistance.

**Table 2:** Scenario and teaching approach of three groups.

**Group**	**Experimental group A**	**Experimental group B**	**Control group**
Teaching method	Project-based learning method	Project-based learning method	Traditional teaching
Teaching time	8 weeks 960 min	8 weeks 960 min	8 weeks 960 min
Teacher	Yes (the same teacher)	Yes (the same teacher)	Yes (the same teacher)
Learning aids	Computer stand-alone environment Graphical program module Scratch	Tablet environment The graphical program module combined with tactile NUWA robotics	Traditional classroom environment Scratch, the unplugged teaching group

One week after the end of the teaching, a post-test (O_2_) was conducted on the three groups of students with the modified Bebras questions (a modified version A) to understand whether different teaching methods of computational thinking could help students improve their computing thinking capabilities. It was further meant to explore whether the teaching methods of these three groups showed significant differences to improve students' computing thinking capabilities.

At the end of the semester, revised Bebras questions (a modified version B) were further modified to implement delayed post-tests (O_3_) on three groups of students. These evaluated the maintenance status for students using their improved computing thinking capabilities, as affected by the different teaching methods of computational thinking after half a semester. It was also further meant to evaluate the different degrees of improved computational thinking ability in the three groups of students, as affected by teaching methods. This study didn't involve any human subject research, and all participants are voluntary. Regardless of control group or experimental group, the informed consent of the procedures and possible risks were obtained in the form of written and witnessed documentation from the participant. Additionally, we ensure the anonymity of research participants is maintained in subsequent reports.

## Results

### Learning Achievement

Before the experiment, a test of Bebras questions (O1) was conducted to understand the differences in computational thinking among the three groups. The results are presented in Table 3.

**Table 3:** Pre-test results of the computational thinking.

**Group**		***N***	**Mean**	**SD**
(EA)	Experimental group A	35	46.49	24.69
(EB)	Experimental group B	35	48.51	33.04
(C)	Control group	35	53.69	27.11

After the learning activity, the three groups of students took the post-test. The pre-test scores were regarded as the covariance for analysis of covariance (ANCOVA) to delete the effects of pretests on learning outcome. The homogeneity of the regression coefficient was tested, which revealed that interaction F between the covariance was 0.275 (*p* > 0.05). This confirms the hypothesis of homogeneity of the regression coefficient.

Table 4 shows the ANCOVA result on the post-test scores of the three groups. The means and standard deviations of the post-test scores were 202.69 and 31.13 for experimental group A, 207.46 and 28.67 for experimental group B, and 189.60 and 26.10 for the control group. The post-test scores of the three groups are significantly different with *F* = 4.12 (*p* < 0.05). The pairwise comparisons show a significant difference between experimental group B and the control group. Thus, students in experimental group B had significantly better learning achievement than the students in the control group.

**Table 4:** ANCOVA results of learning achievement on the post-test scores of the three groups.

	**Group**	***N***	**Mean**	**S.D**.	***F***	**Partial eta squared**	**Pairwise comparisons**
(EA)	Experimental group A	35	202.69	31.13	4.12^*^	0.075	(EB) > (C)^*^
(EB)	Experimental group B	35	207.46	28.67			
(C)	Control group	35	189.60	26.10			
	Total number of students	105	199.91	29.42			

* *p < 0.05*.

Table 5 shows the ANCOVA result on the follow-up test scores of the three groups. The means and standard deviations of the post-test scores were 202.69 and 31.13 for experimental group A, 207.46 and 28.67 for experimental group B, and 189.60 and 26.10 for the control group. The post-test scores of the three groups are significantly different with *F* = 4.12 (*p* < 0.05). Pairwise comparisons show a significant difference between experimental group B and the control group. Thus, students in experimental group B had significantly better learning achievement than students in in the control group.

**Table 5:** ANCOVA results of learning achievement on the followup-test scores of the three groups.

	**Group**	***N***	**Mean**	**S.D**.	***F***	**Partial eta squared**	**Pairwise comparisons**
(EA)	Experimental group A	35	185.89	32.18	3.24^*^	0.060	(EB) > (C)^*^
(EB)	Experimental group B	35	190.31	29.48			
(C)	Control group	35	173.91	25.97			
	Total number of students	105	183.37	29.86			

* *p < 0.05*.

### Cognitive Load

Table 6 presents the analysis results of the students' cognitive load. The mean and standard deviation are 3.83 and 1.22 for the control group, 3.97 and 1.34 for experimental group A, and 4.11 and 1.37 for experimental group B. Although both experimental groups had slightly higher mean scores than the control group, the ANOVA result (*F* = 0.42 and *p* > 0.05) showed no significant difference between the three groups, implying that the three groups of students experienced equivalent levels of cognitive load during the learning activity. Moreover, the average cognitive loads of the three groups were not high, implying that project-based learning courses of thinking skills training provide an easy and relaxed way for students to learn. In addition, it is noteworthy that experimental group A had lower average cognitive load than experimental group B, showing that the lead in of the computer-based visual program could have eased the cognitive load of the students using the robot-based visual program to access the computational thinking courses.

**Table 6:** ANOVA result of cognitive load on the three groups.

	**Group**	***N***	**Mean**	**S.D**.	***F***
(EA)	Experimental group A	35	3.97	1.34	0.42
(EB)	Experimental group B	35	4.11	1.37	
(C)	Control group	35	3.83	1.22	

## Discussions and Conclusions

According to the multisensory teaching theory, when courses provide learners of varied learning preference with information through visual, auditory and haptic means, the learners' learning performance will be improved. This paper includes a discussion of the theoretical implications of PBL for the learning performance, supporting applied technology *via* tangible robots based on multisensory-oriented project. We explained how tangible robots can be integrated into project-based learning courses of thinking skills training to improve the learning performance of the computational thinking ability.

The educational implications of this study are far reaching. A learning activity compared the learning performance of students who used the traditional teaching method integrated with paper practice teaching materials, students who used the project-based learning method integrated with the teaching material of computerized visual programs, and students who used the project-based learning method integrated with the teaching material of robotic visual programs.

Experimental results show that the project-based learning method integrated with the teaching material of robotic visual programs approach had significantly better effectiveness in improving students' learning achievements than the traditional teaching method integrated with paper practice teaching materials approach. Analysis of the questionnaire results showed that the proposed learning approach did not increase the students' cognitive burden. In sum, the proposed approach helps students' learning achievement and cognitive load.

Although the experimental results demonstrate the benefits of using a project-based learning method integrated with the teaching material of robotic visual programs, there are some limitations in the present study. For example, although tangible educational robotic, such as the NUWA, are becoming increasingly popular, few students have access to such a learning technology at present. However, researchers have predicted that robots will become a common learning tools in the near future (Benitti, 2012; Taylor and Baek, 2019; Kucuk and Sisman, 2020; Sen et al., 2021), implying that the proposed approach has the potential to become a widely-used learning model. Another limitation of this study is that the learning courses of thinking skills training was conducted with college freshmen. Therefore, one of our future research directions is to extend this study to other subjects and to students of different ages to further evaluate the effectiveness of this approach.

## Data Availability Statement

The original contributions presented in the study are included in the article/supplementary material, further inquiries can be directed to the corresponding authors.

## Author Contributions

M-CH, H-CP, and S-WH: conceptualization. S-WH: methodology, software, and data curation. M-CH and S-WH: validation and writing—review and editing. H-CP: formal analysis. M-CH, M-JH, and S-WC: investigation, resources, and supervision. M-CH and H-CP: writing—original draft preparation. All authors have read and agreed to the published version of the manuscript.

## Funding

This work was supported by the Ministry of Science and Technology, Taiwan under Project Number 108-2511-H-269-001-MY2.

## Conflict of Interest

The authors declare that the research was conducted in the absence of any commercial or financial relationships that could be construed as a potential conflict of interest.

## Publisher's Note

All claims expressed in this article are solely those of the authors and do not necessarily represent those of their affiliated organizations, or those of the publisher, the editors and the reviewers. Any product that may be evaluated in this article, or claim that may be made by its manufacturer, is not guaranteed or endorsed by the publisher.
